# Synthesis of α-*O*- and α-*S*-Glycosphingolipids Related to *Sphingomonous* cell Wall Antigens Using Anomerisation

**DOI:** 10.3390/molecules180911198

**Published:** 2013-09-12

**Authors:** Wayne Pilgrim, Ciaran O’Reilly, Paul V. Murphy

**Affiliations:** 1 School of Chemistry and Chemical Biology, University College Dublin, Dublin 4, Ireland; 2 School of Chemistry, National University of Ireland Galway, University Road, Galway, Ireland

**Keywords:** α-GalCer, anomerisation, glucuronic acid, galacturonic acid, glycoside bond, antigens, NKT cells

## Abstract

Analogues of glycolipids from *Spingomonadacaece* with *O*- and *S*- and *SO*_2_-linkages have been prepared using chelation induced anomerisation promoted by TiCl_4_. Included are examples of the anomerisation of intermediates with *O*- and *S*-glycosidic linkages as well as isomerisation of β-thioglycuronic acids (β-glycosyl thiols). The β-*O*-glucuronide and β-*O*-galacturonide precursors were efficiently prepared using benzoylated trichloroacetimidates. β-Glycosyl thiols were precursors to β-*S*-derivatives. Triazole containing mimics of the natural glycolipids were prepared using CuI promoted azide-alkyne cycloaddition reactions in THF. The glycolipid antigens are being evaluated currently for their effects on iNKT cells.

## 1. Introduction

**Figure 1:**
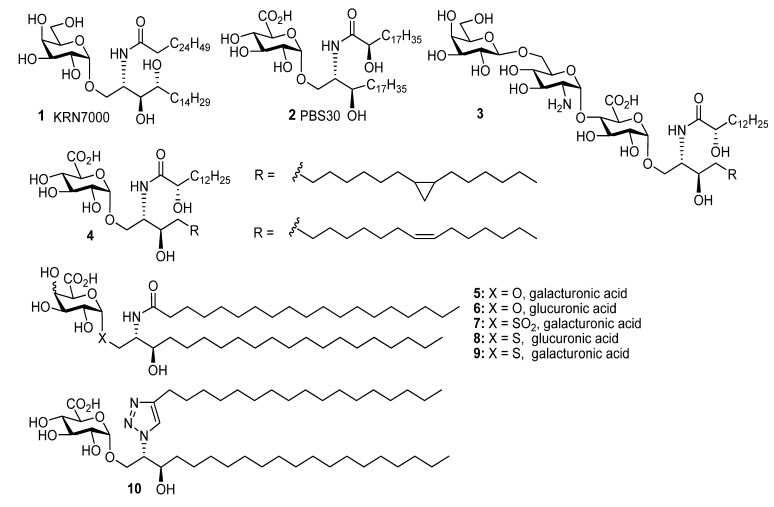
Structures of glycosphingolipids **1**–**10**.

CD1d restricted invariant natural killer T cells (iNKT cells) are a class of lymphocytes activated in response to specific glycolipid antigens. Stimulation of iNKT cells by glycolipids can cause secretion of Th1, Th2, Th17 and Treg cytokines and there could be advantages in clinical therapy for glycolipids to be identified that can induce a bias towards secretion of Th1 or Th2 cytokines. The prototype antigen α-galactosylceramide (α-GalCer, **1**) stimulates iNKT cells to kill tumour cells, release cytokines and activate other cells of the immune system in mice. Consequently there has been interest in evaluating glycolipid antigens with potential as anti-infective agents & vaccine adjuvants [1,2,3,4,5,6,7,8,9,10], and clinical trials of some glycolipids have been undertaken [11] or their preclinical evaluation is advanced [12]. The cell walls of *Sphingomonadacae* bacteria present uronic acid containing glycosphingolipids such **2**–**4** [1,2,3,7] which stimulate iNKT cells. *Sphingomonadacae* aregram-negative bacteria that can cause infection in humans. While monosaccharide and higher order saccharide (e.g., **3**, Figure 1) antigens are known in these bacteria, it seems that the most potent stimulators of the iNKT cells are monosaccharides [7]. Such bacterial glycolipids have been shown to induce septic shock and bacterial clearance in infected mice, demonstrating that glycolipids stimulate an innate-type immune response to gram negative bacteria [2,3]. The *Sphingomonadacae* glycolipids contain glucuronic acid or galacturonic acid residues which are α-*O*-linked to sphinganine derivatives (Figure 1), and they are structurally related to α-GalCer **1**. The α-glycosidic linkage seems advantageous in generating highly potent stimulatory properties. In this article we present a full account of work on the synthesis of α-*S*-, *SO*_2_- and *O*-linked glycolipids **5**–**10** which are based on glucuronic acid and galacturonic acid. This article supplements two preliminary communications [13,14] where we have outlined how chelation induced anomerisation [15,16,17,18] can be used for the preparation of *S*- and *O*- glycolipids relevant to biology and medicine. Herein we provide additional examples and more detail regarding our initial efforts in this area.

## 2. Results and Discussion

**Figure 2:**
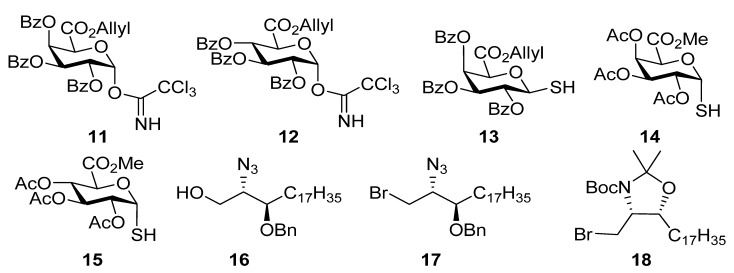
Structures of key building blocks.

The structures of the key building blocks **11**–**18** used in the synthesis of the glycosphinoglipid antigens **5**–**10** are shown in Figure 2. Highly stereoselective glycosidations of uronic acids can be difficult to achieve and we had observed high selectivity in favour of the α-anomer in anomerisation of simple α-*O* and α-*S*-glucuronic acid derivatives. The synthesis of this type of glycolipid provided a more challenging biologically relevant target with a view to testing the scope of the Lewis acid catalysed anomerisation reactions. The overarching aim of this research was thus to establish whether TiCl_4_ promoted anomerisation reactions could be utilized in synthesis of these biologically interesting compounds. The planned strategy involved preparation of potential α-*O* or α-*S* glycoside precursors to the glycolipids and to investigate their subsequent anomerisation and then work out the remaining steps to give the unprotected glycolipids. The synthesis of **6**, **7** and **9** has been previously detailed [13,14] and we supplement the reports on the preparation of those compounds with details of the preparation of **5**, **8** and **10**. We have included comparisons of yields and stereoselectivities of the various steps from the previous reports for completeness. In recent research, a triazole was incorporated into α-GalCer analogues as an isostere of the sphingosine amide. This conferred interesting and desirable biological properties, as triazole analogues with a long alkyl chains had potent stimulatory effects on cytokine production and showed a stronger Th2 cytokine response than found for α-GalCer [19]. Therefore, we included the preparation of such a triazole analogue containing a uronic acid **10** as part of this research work. The research therefore began with the synthesis of *O*-glycosides from the trichloroacetimidates **11** and **12** which were used in conjunction with alcohol **16**. In addition we used the thioglycuronic acid derivatives **13**–**15** which were used jointly with the bromides **17** and **18** in the generation of *S*-linked glycolipids.

**Scheme 1:**
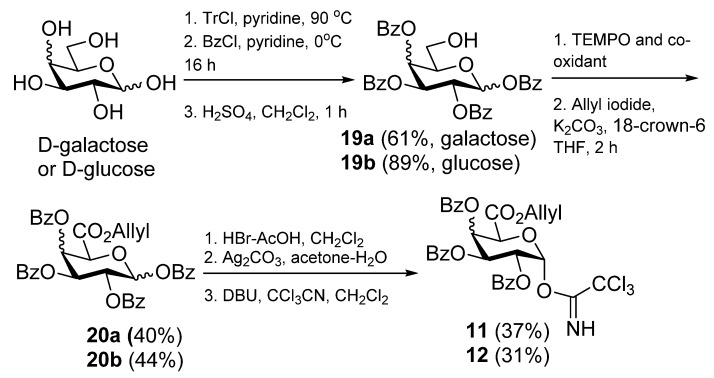
Synthesis of trichloroacetimidates.

The preparation of the trichloroacetimidates **11** and **12** is summarized in Scheme 1. The regioselective protection of d-galactose and d-glucose using trityl chloride in pyridine was followed by benzoylation in the presence of pyridine. Subsequent hydrolysis of the trityl group using sulfuric acid in dichloromethane gave the alcohols **19a** and **19b**. Oxidation of the glucose derivative **19b** with TEMPO-NaOCl proceeded satisfactorily to give the glucuronic acid. However, these conditions were not successful for the oxidation of the galactose derivative **19a**. Nevertheless, the reaction of **19a** with TEMPO and BAIB as co-oxidant proceeded smoothly to give the galacturonic acid precursor of **20a**. Esterification of the acids via the generation of the carboxylates and their reaction with allyl iodide gave **20a** and **20b**. Formation of the ester **20a** via the acid chloride, synthesised from reaction of the acid with oxalyl chloride and DMF in CH_2_Cl_2_ followed by addition of allyl alcohol was also investigated. This route provided the allyl ester **20a** in similar yield (40% *vs.* 44%). Next the glycosyl bromides were formed by treatment of **20a** and **20b** with HBr-AcOH in dichloromethane. Reaction of the isolated bromides with silver carbonate in water and acetone followed by reaction with trichloroacetonitrile in the presence of DBU gave the glycosyl donors **11** and **12**.

**Scheme 2:**
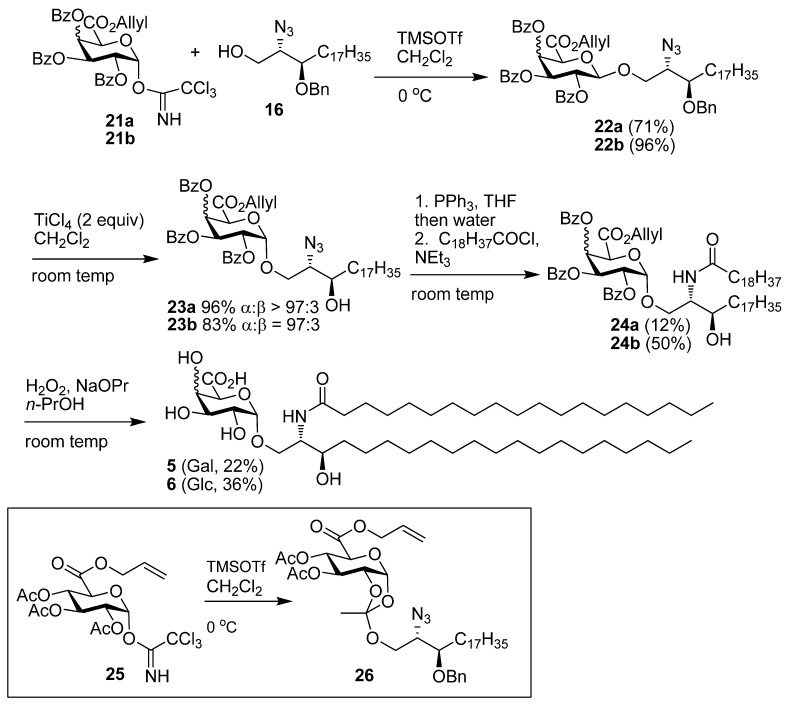
Synthesis of **5** and **6**.

With the synthesis of both donors **11** and **12** achieved, the glycoside coupling reactions and subsequent chelation induced anomerisation were investigated (Scheme 2). The optimal strategy was to first prepare the β-glycoside **22** by coupling the trichloroacetimidates **11** and **12** with acceptor **16** [13] using TMSOTf. The benzoylated donor **21b** was superior to the related acetylated donor **25**. Glycosidation of **25** with **16** under the same conditions as reaction with **12** led to the isolation of the corresponding orthoester **26**. Evidence for the orthoester was obtained by NMR analysis which, for example, showed an anomeric proton at δ 5.74 ppm (*J*_1,2_ 5.2 Hz) in the ^1^H-NMR spectrum. The next step was the application of TiCl_4_ to promote anomerisation. This reaction using 2 equiv of TiCl_4_ did proceed in a satisfactory manner to generate the required α-anomers **23a** and **23b** from the β-glycoside precursors. The yields and stereoselectivities were high (97:3 or greater) for these reactions. The Lewis acid conveniently removed the benzyl group from the sphinganine residue under these conditions. It was found that anomerisation proceeded faster than the cleavage of the benzyl ether. Thus, the benzyl protected α‑anomer could be isolated if required. With **23** in hand, the azide groups were reduced using the Staudinger reaction and subsequent coupling of the amine with nonadecanoyl chloride in the presence of triethylamine gave amides **24**. Although the benzoates were advantageous in the glycosidation reaction, they were not easily removed in the final step. Reaction with methoxide in methanol led to elimination of benzoic acid and formation of an unsaturated product whereas reaction with K_2_CO_3_ in methanol and water led to removal of the allyl ester but under these conditions the benzoyl protecting groups were found to be stable. Finally the removal of all the protecting groups was successfully carried out using hydroperoxide ion generated from hydrogen peroxide in *n*-propanol using sodium propoxide as base. These conditions were used in order to maximise solubility of the glycolipid by using a more hydrophobic solvent than methanol or ethanol. Centrifugation of the product mixture and precipitation of the insoluble glycolipids and subsequent water washing was used to isolate the products **5** and **6**. Success of the centrifugation method for purification was dependant on the relative solubility of each glycolipid. Therefore the amount of solvent used in each reaction had to be adjusted to match the solubility of each glycolipid. The deprotection of **24a** was carried out in less solvent (3 mL for 10 mg precursor) than **24b** (5 mL for 10 mg precursor) in order to ensure that precipitation of the glycolipid would occur. The low yields in the final step are generally attributed to the product not precipitating and to some of the precipitate being re-dissolved in the water washing step.

**Scheme 3:**
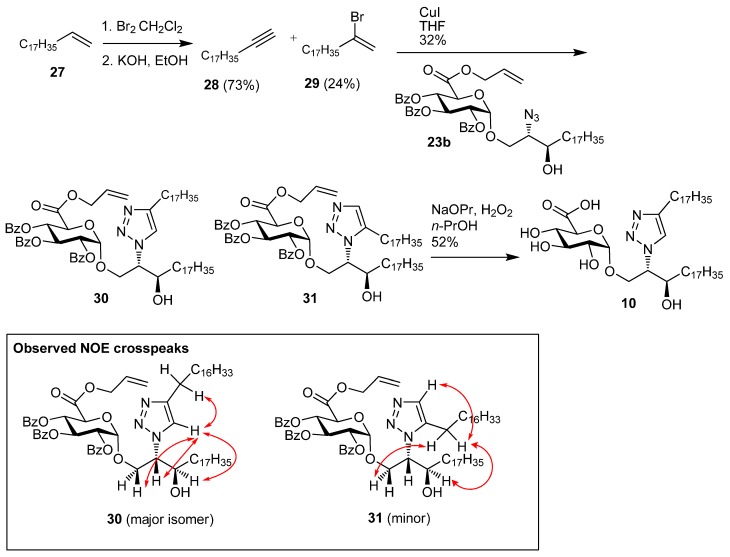
Synthesis of **10**.

Given that the azide **22b** was available its conversion into the triazole-containing glycolipid **10** was also investigated. The synthesis of 1-nonadecyne **28** from 1-nonadecene **27** used a method related to an established procedure for the synthesis of 1-heptadecyne [20]. Thus, the addition of bromine followed by didehydrohalogenation with KOH in EtOH gave **28** (73%) as well as 2-bromononadec-1-ene **29** (24%) (Scheme 3). Initial attempts at promoting the copper catalysed azide-alkyne cycloaddition [21] were investigated by coupling **23b** and **28**. However reactions using CuSO_4_ and sodium ascorbate in various mixtures of THF, H_2_O, and *^t^*BuOH were not successful [22,23]. This was attributed to the low solubility of **28**. In contrast the use of CuI in THF [24] led to a successful coupling of **23b** and **28** and gave an 86:14 mixture of the triazoles **30** and **31** (32%). The 1,4 configuration was assigned to the major product (**30**) on the basis of both NOESY and ROESY analysis. The 1,4-regioisomer (**30**) showed interactions between the triazole proton with a number of protons on the dihydroceramide chain (see Scheme 3 for NOE correlations) indicating that they are in close proximity. The triazole proton of the minor regioisomer **31** is more remote from the dihydroceramide chain, and as would be expected for this isomer there was not any NOE cross-peaks observed between this proton and the dihydroceramide chain. The regioselectivity of this Cu(I) promoted cycloaddition reaction was not as high as that reported for the previously synthesised triazole containing glycolipids, were the 1,4 isomer was not reported [19]. The protecting groups were removed from **30/31** to give a mixture of triazoles (52%) where **10** was the major component (ratio of 1,4 to 1,5-isomer, 88:12).

**Scheme 4:**
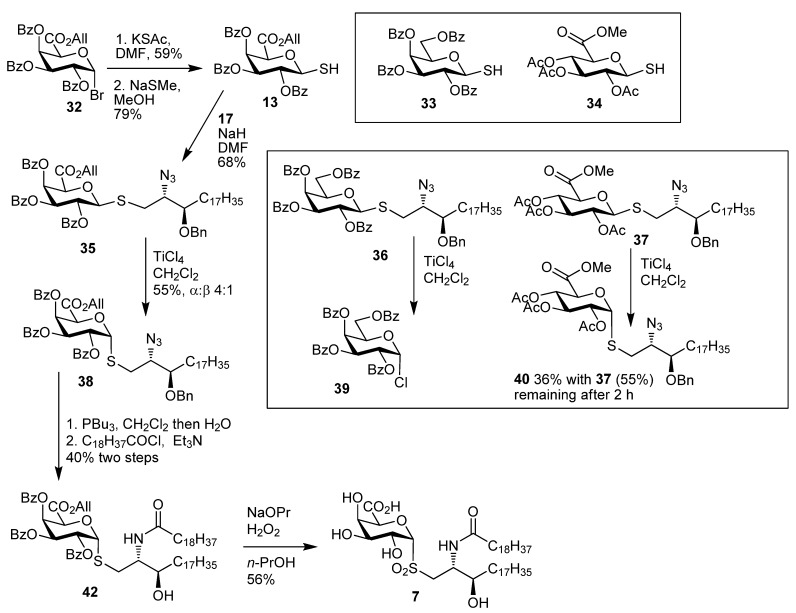
Synthesis of **7**.

The synthesis of *S*-glycoside analogues of natural bioactive *O*-glycosides has been of interest as potential glycomimetics [25,26,27]. *S*-Glycosides are apparently more stable *in vivo* than native *O*-glycosides and also there is the possibility that they are more immunogenic, which is relevant to vaccine development [28]. Kinetic studies on the anomerisation of *S*-butyl and *O*-butyl glycosides, as well as the formation of **23a** and **23b**, indicated that anomerisation of *S*-glycolipids would be feasible [14]. As reviewed by Pachamuthu and Schmidt [27], the synthesis of thioglycolipids can be approached by preparation of a lipid containing thiol [29], which is then reacted with a glycosyl halide. Alternatively a glycosyl thiol can be generated, which is then reacted with a lipid which has an appropriate leaving group. We investigated the former approach for the glycosphingolipids, however in our hands this was not successful. In light of this, the formation of the thioglycoside by direct anomeric alkylation of a glycosyl thiol with a dihydroceramide moiety containing a suitable leaving group was investigated. Thiol **13** (79%) was synthesised from the glycosyl bromide **32** through its reaction with KSAc and subsequent selective deprotection using NaSMe (Scheme 4). Initially the direct anomeric alkylation to give **35** was attempted using the mesylate of the alcohol **16**. However efforts to use this mesylate led to recovery of both starting materials, with no thioglycoside formation occurring. Reaction of the mesylate at 90 °C with KBr in DMF gave the bromide **17** (93%). The coupling of this bromide with the thiol **13** was carried out using NaH as base, and this gave the β-thioglycoside **35**. It is worth noting that this alkylation reaction should not be carried out using excess NaH as this gave rise to the previously mentioned elimination of benzoic acid and formation of an unsaturated saccharide.

With **35** in hand, the thioglycosides **36**–**37** were also prepared, and their anomerisations investigated. Anomerisation of **37** using TiCl_4_ in CH_2_Cl_2_ led to the formation of **40** (36%) with **37** also being recovered (53%) after 2 h. When longer reaction times were investigated decomposition occurred. The galactose derivative **36** was prepared in high yield from the bromide **17** (93%) and the corresponding thiol. However anomerisation, using TiCl_4_ in CH_2_Cl_2_ led to the α-chloride **39** (21%) being formed, while **36** was also recovered in substantial amounts after 24 h at room temp. A prolonged reaction with TiCl_4_ (72 h) or the use of SnCl_4_ led to the α-chloride **39** as the sole product. Formation of **39** is consistent with activation of the thioglycoside. It is possible that the presence of the azide and the OBn group in the lipid is chelating efficiently to the Lewis acid leading to activation of the thioglycoside which is followed by substitution with a chloride from the Lewis acid. In contrast the anomerisation of **35** was more successful and is explained by chelation to the C-5 carbonyl group and the pyranose oxygen atom to the Ti(IV) species, which leads to endocyclic cleavage and anomerisation rather than thioglycoside activation and glycosyl chloride formation. This was supported by kinetics studies of simple galacturonic acid and galactose substrates which we have described previously [18]. Hence the anomerisation reaction of **35** carried out with TiCl_4_ in dichloromethane gave the α-anomer **38** in 55% yield after chromatography. The stereoselectivity (4:1) in the anomerisation reaction was not as high as for anomerisation of the *O*-galacturonide. This was presumed to be because of a weaker anomeric effect for sulfur when compared with oxygen which could be due to the sulfur being less electron withdrawing than oxygen or due to steric reasons where the larger sulfur atom shows a higher preference for the equatorial position or due to an equilibrium not being attained. Although a mixture of anomers was formed they could be separated by column chromatography to give **38** in an isolated 55% yield. Reduction of the azide and coupling with nonadecanoyl chloride was carried out as described earlier to provide **42** in 40% yield. Treatment of **42** with NaOPr-H_2_O_2_-PrOH led to the removal of the protecting groups with concomitant oxidation of the anomeric sulfur atom to give the sulfone **7** (56%).

**Scheme 5:**
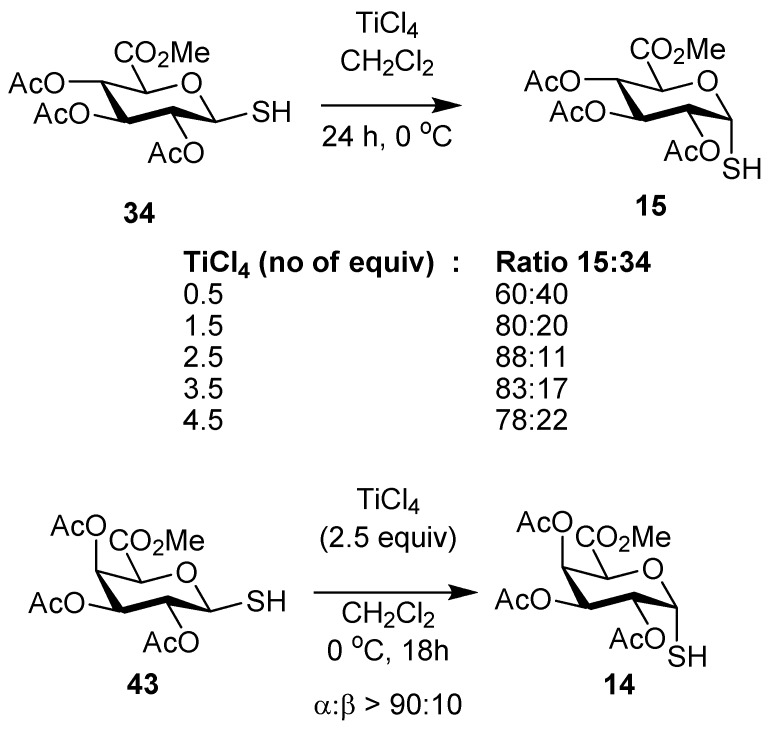
Synthesis of α-thioglycopyuranuronic acid derivatives.

The synthetic strategy to the *S*-glycolipids was next revised, given that the final deprotection led to sulfur oxidation. We thus investigated the synthesis of glycosyl thiols **14** and **15** and envisaged they could be used in conjunction with the bromide **18**. The glycosyl thiols **34** and **43** which had the β-configuration were prepared as described previously [30]. Our earlier work indicated that the stereoselectivity of the anomerisation reaction of simple *O*- and *S*-glycosides could be influenced by the relative amount of TiCl_4_ that would be used. The concentration of TiCl_4_ used was varied from 0.5 equivalents to 4.5 equivalents during an investigation of the anomerisation reactions of **34**. The α-thiol **15** was formed and the use of 2.5 equivalents of TiCl_4_ gave the optimum proportion of **15**:**34** (8:1). The formation of the α-thiogalacturonate **14** proceeded from **43** with good selectivity (>9:1) for **15** under the same conditions. The α:β ratio was not just dependent on TiCl_4_ concentration but also on temperature, as higher ratios were observed for reactions at 0 °C as compared to reactions at room temp. The α:β ratio was found to be scale dependant. For the formation of **15** on a 100 mg scale, the α:β ratio was > 97:3 and was 9:1 when carried out on a one gram scale. This difference may be due to a greater exotherm on the larger scale which led to warming of the reaction mixture with a consequent increase in proportion of the β-anomer. An alternative explanation is that this reaction may not have attained equilibrium on the larger scale. Nevertheless, the transformation was synthetically useful. The presence of the C-6 carbonyl was found to be critical in order for the anomerisation of the thiol to proceed efficiently. The anomerisation reaction of the corresponding 2,3,4,6-tetra-*O*-acetyl β-d-thiogalactopyranose, for example, did not proceed under identical conditions, supporting the notion that chelation by both the C-5 carbonyl and ring oxygen to the Lewis acid is necessary for efficient anomerisation (Scheme 5).

**Scheme 6:**
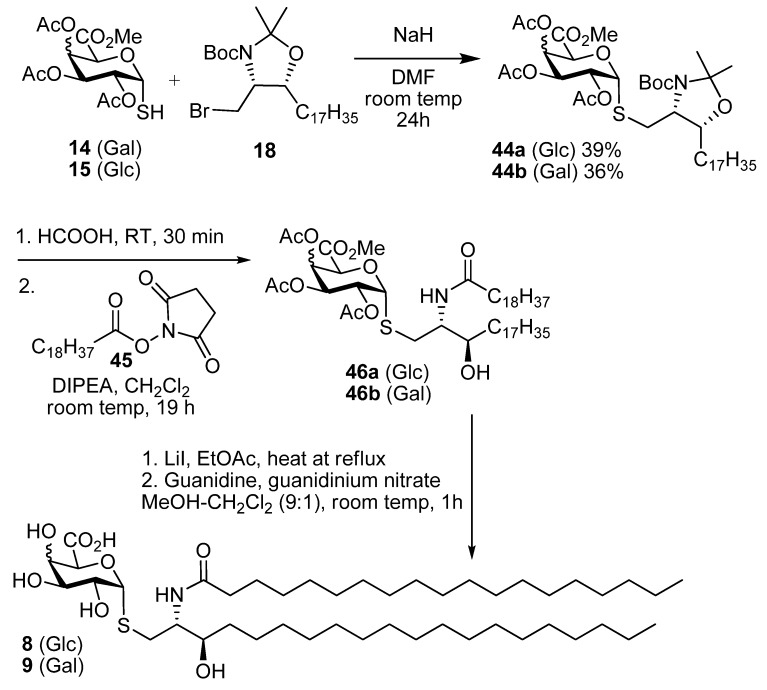
Synthesis of **8** and **9**.

With **14** and **15** available the completion of the synthesis of the *S*-glycolipid could be achieved (Scheme 6). The coupling of the thiols **14** and **15** was brought about using NaH (<1 equiv) and **18** to give the protected lipids **44a** and **44b** in 36%–39% yield. The use of other bases for this reaction led to lower yields or only trace amounts of **44a** and **44b**. Also the addition of additives such as tetra-*N*-butyl ammonium iodide did not lead to an improvement in yield. The use of a Mitsunobu condensation reaction was also investigated. In this case the thiol could be reacted with the alcohol precursor to **18** using 1,1'-(azodicarbonyl)dipiperidine and trimethylphosphine [31] and this approach gave similar yields to the *S*-alkylation of **18**. The protected thioglycolipids **44a** and **44b** were then treated with formic acid [32] for 30 min to remove both the oxazolidine and Boc groups and this gave the aminoalcohol intermediates in 85% yield. Reaction of this aminoalcohol with the succinate **45** [33] in dichloromethane gave the amides **46a** and **46b** (60%); these yields that were better than those from the corresponding acid chloride. Finally, the removal of the protecting groups from the uronic acid residues gave **8** and **9**. This was achieved in two steps. The methyl ester was removed using LiI in EtOAc [34], and a subsequent reaction with guanidine and guanidinium nitrate [35] in methanol-dichloromethane gave **8** and **9**.

## 3. Experimental

### General

Optical rotations were determined with a Perkin-Elmer 343 model polarimeter at the sodium D line at 20 °C. ^1^H-NMR spectra and and ^13^C-NMR were recorded at the frequencies stated. Chemical shifts are reported relative to internal Me_4_Si (δ 0.0) in CDCl_3_ or CDCl_3_-MeOD, or HOD for D_2_O (δ 4.84) or CD_2_HOD (δ 3.31) for ^1^H and Me_4_Si in CDCl_3_ (δ 0.0) or CDCl_3_ (δ 77.0) or CD_3_OD (δ 49.05) for ^13^C. ^1^H- NMR signals were assigned with the aid of COSY. ^13^C-NMR signals were assigned with the aid of DEPT, HSQC and/or HMBC. Coupling constants are reported in hertz. The IR spectra were recorded as thin films between NaCl plates or with an ATR attachment. Low and high resolution mass spectra were measured on either a micromass VG 70/70H or VG ZAB-E or Waters LCT premiere XE spectrometers and were measured in positive and/or negative mode. Thin layer chromatography (TLC) was performed on aluminium sheets precoated with silica gel and spots visualized by UV and charring with H_2_SO_4_-EtOH (1:20) or cerium molybdate. Flash chromatography was carried out with silica gel 60 (0.040–0.630 mm). Chromatography solvents were used as obtained from suppliers. CH_2_Cl_2_, MeOH, and THF reaction solvents were dried using a Pure Solv™ Solvent Purification System and acetonitrile, DMF, pyridine, and toluene were used as purchased from Sigma-Aldrich. The experimental details for the preparation of **6**, **7**, **9**, **13**, **14**, **16**–**18**, **19b**–**24b**, **35**, **38**, **42**, **43**, **44** and **46b** and their analytical data have been reported previously [13,14].

*1,2,3,4-Tetra-O-benzoyl-α-D-galactopyranuronic acid, allyl ester* (**20a**). d-Galactose (5.4 g, 0.03 mol) and trityl chloride (17.0 g, 0.04 mol) were dried under diminished pressure for 3 h. The mixture was taken up in pyridine (80 mL) and heated at 90 °C for 16 h, then The solution was then cooled to 0 °C and benzoyl chloride (17 mL, 0.15 mol) was added slowlyand he reaction mixture was then allowed to attain room temp, stirred for 20 h, and the mixture was then diluted with CH_2_Cl_2_ and washed with water, 2 M HCl, satd aq NaHCO_3_, brine, dried over Na_2_SO_4_ and the solvent was removed under diminished pressure. The resulting syrup was dissolved in CH_2_Cl_2_-MeOH (1:2, 150 mL) and conc. H_2_SO_4_ (15 mL) was added. The mixture was stirred for 1 h at room temp and then washed with water, satd aq NaHCO_3_, brine, and dried over Na_2_SO_4_, and the solvent was removed under diminished pressure. Flash chromatography (petroleum ether-EtOAc, 4:1) gave **19a** as a white solid and a mixture of anomers (10.9 g, 61%). The NMR data (^1^H and ^13^C) for **19a** were in agreement with data reported in the literature [36]. To **19a** (10.0 g, 0.017 mol) in CH_2_Cl_2_ (100 mL) and H_2_O (40 mL) were added TEMPO (0.5 g) and BAIB (16.2 g, 0.05 mol). The mixture was stirred vigorously for 2 h and satd Na_2_S_2_O_3_ (40 mL) was then added and the resulting mixture stirred for 15 min. The layers were separated and the aq phase acidified with 1M HCl and extracted with CH_2_Cl_2_ (×3). The combined organic layers were dried over Na_2_SO_4_, and the solvent was removed under diminished pressure to give the galacturonic acid intermediate (9.5 g, 91%). The resulting solid was dissolved in THF (100 mL), to this K_2_CO_3_ (115 mg, 0.84 mmol), 18-crown-6 (10 mg) and allyl iodide (140 mg, 0.84 mmol, 80 µL) were added. The reaction was stirred in the dark for 16 h at room temp. The reaction mixture was diluted with Et_2_O, washed with water, sodium thiosulfate, water, brine, dried over MgSO_4_. The solvent was then removed under reduced pressure and then flash chromatography (EtOAc-cyclohexane 1:4) of the residue gave **20a** (484 mg, 44%) as a white solid; R_f_ 0.53 (EtOAc-cyclohexane 2:5); [α]_D_ +87 (*c* 4.7, CHCl_3_); IR (film) cm^−1^: 3065, 2930, 1733, 1451, 1263, 1092, 1069; ^1^H-NMR (CDCl_3_, 400 MHz ): δ 8.05 (4H, m, aromatic H), 7.89 (2H, dd, *J =* 7.2 Hz, *J =* 1.3 Hz, aromatic H), 7.83 (2H, dd, *J =* 7.2, *J =* 1.3 Hz, aromatic H), 7.61 (1H, tt, *J =* 7.5 Hz, *J =* 1.2 Hz, aromatic H), 7.56 (1H, tt, *J =* 7.4, *J =* 1.2 Hz, aromatic H), 7.45 (6H, m, aromatic H), 7.30 (4H, m, aromatic H), 6.28 (2H, m, H-1, H-4), 6.01 (1H, dd, *J =* 10.3 Hz, *J =* 8.3 Hz, H-2), 5.79 (1H, dd, *J =* 10.3 Hz, *J =* 3.5 Hz, H-3), 5.72 (1H, ddt, *J =* 17.2 Hz, *J =* 10.3 Hz, *J =* 6.1 Hz, C*H*=CH_2_), 5.22 (1H, dd, *J =* 17.2 Hz, *J =* 1.3 Hz, CH=C*H_2_*), 5.07 (1H, *J =* 10.3 Hz, *J =* 1.0 Hz, CH=C*H_2_*), 4.86 (1H, d, *J =* 1.5 Hz, H-5), 4.59 (2H, m, OC*H_2_*CH=CH_2_); ^13^C-NMR (CDCl_3_, 100 MHz): δ 165.4, 165.2, 165.1, 165.0 164.7 (each C=O), 133.8, 133.6, 133.4 (2s), 130.7, 130.3, 130.1, 129.8, 129.7, 128.8, 128.7, 128.6, 128.5, 128.4 (3s), 128.3 (each C or CH), 120.0 (alkene CH_2_) 92.6 (C-1), 73.7 (C-5), 71.2 (C-3), 69.0 (C-4), 68.2 (C-2), 66.7 (O*C*H_2_CH=CH_2_); ESI-HRMS calcd for C_37_H_30_O_11_Na 673.1686, found *m/z* 673.1714 [M+Na]^+^.

*2,3,4-Tri-O-benzoyl-1-deoxy-1-(2,2,2-trichloro-1-iminoethoxy)-α-d-galactopyranuronic acid,allyl ester* (**11**). Allyl ester **20a** (325 mg, 0.50 mmol) was dissolved in CH_2_Cl_2_ (3 mL) and cooled to 0 °C. To this HBr 33% in AcOH (2 mL) was added and the reaction stirred at room temp for 3 h. The reaction was quenched with satd NaHCO_3)_, washed with water, brine, dried over MgSO_4_, and the solvent removed under reduced pressure to give the intermediate bromide (237 mg, 78%); ^1^H-NMR (CDCl_3_, 400 MHz): δ 7.99 (4H, d, *J =* 7.4 Hz, aromatic H), 7.82 (2H, d, *J =* 7.4 Hz, aromatic H), 7.61 (1H, t, *J =* 7.4 Hz, aromatic H), 7.54 (1H, t, *J =* 7.4 Hz, aromatic H), 7.46 (3H, m, aromatic H), 7.39 (2H, t, *J =* 7.8 Hz, aromatic H), 7.28 (2H, t, *J =* 7.8 Hz, aromatic H), 7.02 (1H, d, *J =* 3.9 Hz, H-1), 6.32 (1H, dd, *J =* 3.2 Hz, *J =* 1.3 Hz, H-4), 6.05 (1H, dd, *J =* 10.5 Hz, *J =* 3.2 Hz, H-3), 5.75 (1H, ddt, *J =* 17.1 Hz, *J =* 10.5 Hz, *J =* 6.1 Hz, C*H*=CH_2_), 5.66 (1H, dd, *J =* 10.5 Hz, *J =* 3.9 Hz, H-2), 5,24 (1H, dd, *J =* 17.1 Hz, *J =* 0.8 Hz, *J =* CH=C*H_2_*), 5.17 (1H, d, *J =* 1.3 Hz, H-5), 5.10 (1H, d, *J =* 10.3 Hz, CH=C*H_2_*), 4.60 (2H, m, OC*H_2_*CH=CH_2_); ^13^C-NMR (CDCl_3_, 100 MHz): δ 165.4, 165.3, 165.0, 164.9 (each C=O), 133.9, 133.7, 133.4, 130.5, 130.0, 129.9, 129.7 (3s), 128.6, 128.5, 128.4, 128.3 (each C or CH), 120.2 (alkene CH_2_), 87.4 (C-1), 72.9 (C-5), 68.7 (C-4), 68.6 (C-3), 67.9 (C-2), 66.8 (O*C*H_2_CH=CH_2_). This bromide (237mg, 0.39 mmol) was dissolved in acetone (18 mL) and H_2_O (2 mL). To this Ag_2_CO_3_ (64 mg, 0.23 mmol) was added and the reaction stirred in the dark at room temp for 24 h. The reaction mixture was filtered through celite, which was rinsed with CH_2_Cl_2_. The filtrate was washed with water, brine, dried over MgSO_4_, and the solvent removed under reduced pressure. Flash chromatography (EtOAc-cyclohexane 2:5) gave the hemiacetal (164 mg, 77%) as a colourless oil; R_f_ 0.18 (EtOAc-cyclohexane 2:5); IR (film) cm^−1^: 3446, 3069, 2961, 1730, 1264, 1094, 1070, 1026; ES-HRMS calcd for C_30_H_27_O_10_ 547.1604, found *m/z* 547.1612 [M+H]^+^; NMR data for the α anomer: ^1^H-NMR (CDCl_3_, 400 MHz): δ 8.00-7.26 (15H, ms, aromatic H), 6.30 (1H, dd, *J =* 3.5 Hz, *J =* 1.6 Hz, H-4), 6.08 (1H, dd, *J =* 10.7 Hz, *J =* 3.5 Hz, H-3), 5.96 (1H, t, 3.6 Hz, H-1), 5.70 (2H, overlapping signals, H-2 & C*H*=CH_2_), 5.22 (1H, dd, *J =* 17.2 Hz, *J =* 1.3 Hz, CH=C*H_2_*), 5.18 (1H, d, *J =* 1.5 Hz, H-5), 5.06 (1H, dd, *J =* 10.4 Hz, *J =* 1.1 Hz, CH=C*H_2_*), 4.62 (1H, dd, *J =* 12.5 Hz, *J =* 6.5 Hz, OC*H_2_*CH=CH_2_), 4.55 (1H, dd, *J =* 12.5 Hz, *J =* 6.3 Hz, OC*H_2_*CH=CH_2_), 4.16 (1H, d, *J =* 3.6 Hz, OH); ^13^C-NMR (CDCl_3_, 100 MHz): δ 167.3, 165.9, 165.6, 165.2 (each C=O), 133.5, 133.4, 133.1, 130.7, 129.9, 129.8 (2s), 129.7, 129.1 (2s), 129.0, 128.5, 128.4, 128.2, 119.9 (alkene CH_2_), 91.2 (C-1), 69.9 (C-4), 68.8 (2s, C-2 & C-5), 67.7 (C-3), 66.5 (O*C*H_2_CH=CH_2_); selected NMR data for the β anomer: ^1^H NMR (CDCl_3_, 400 MHz): δ 6.22 (1H, dd, *J =* 3.4 Hz, *J =* 1.5 Hz, H-4), 5.29 (1H, s), 4.68 (1H, d, *J =* 1.5 Hz), 4.30 (1 H, d, *J =* 8.3 Hz, OH); ^13^C NMR (CDCl_3_, 100 MHz): δ 166.6, 165.9, 165.5, 165.1 (each C=O), 120.0 (alkene CH_2_), 96.1 (C-1), 73.0 (C-5), 71.4 (C-2), 70.7, 69.0, 66.7 (O*C*H_2_CH=CH_2_). This hemiacetal (166 mg, 0.30 mmol) was dissolved in CH_2_Cl_2_ (6 mL) and cooled to 0 °C. To this trichloroacetonitrile (0.3 mL, 3.0 mmol) and DBU (5 drops) were added, and the mixture was stirred at 0 °C for 4 h. The solvent was removed under reduced pressure to a volume of 2 mL and flash chromatography (EtOAc-cyclohexane 1:4) then gave **11** (133 mg, 63%) as a yellow oil; R_f_ 0.34 (EtOAc-cyclohexane 2:5); IR (film) cm^−1^: 3328, 3072, 2958, 1735, 1677, 1452, 1265, 1106, 1068, 1027, 709; ^1^H-NMR (CDCl_3_, 500 MHz): δ 8.69 (1H, s, NH), 8.02 (2H, d, *J =* 7.2 Hz, aromatic H), 7.94 (2H, *J =* 7.2 Hz, aromatic H), 7.82 (2H, d, *J =* 7.2 Hz, aromatic H), 7.60 (1H, t, *J =* 7.5 Hz, aromatic H), 7.46 (4H, m, aromatic H), 7.34 (2H, t, *J =* 7.8 Hz, aromatic H), 7.27 (2H, t, *J =* 7.8Hz, aromatic H), 7.05 (1H, d, *J =* 3.5 Hz, H-1), 6.37 (1H, dd, *J =* 3.3 Hz, *J =* 1.4 Hz, H-4), 6.10 (1H, dd, *J =* 10.7 Hz, *J =* 3.3 Hz, H-3), 5.96 (1H, *J =* 10.7 Hz, *J =* 3.5 Hz, H-2), 5.75 (1H, ddt, *J =* 17.1 Hz, *J =* 10.4, *J =* 6.1 Hz, C*H*=CH_2_), 5.22 (1H, dd, *J =* 17.1 Hz, *J =* 1.2 Hz, CH=C*H_2_*), 5.11 (1H, d, *J =* 1.4 Hz, H-5), 5.07 (1H, dd, *J =* 10.4 Hz, *J =* 0.7 Hz, CH=C*H_2_*), 4.62 (1H, dd, *J =* 12.7 Hz, *J =* 6.1 Hz, OC*H_2_*CH=CH_2_), 4.56 (1H, dd, *J =* 12.7 Hz, *J =* 6.1 Hz, OC*H_2_*CH=CH_2_); ^13^C-NMR (CDCl_3_, 125 MHz): δ 165.6, 165.4, 165.4, 165.0 (each C=O), 160.2 (C=N), 133.6, 133.5, 133.3, 130.7, 129.9, 129.8, 129.7, 128.8 (2s), 128.6, 128.5, 128.4, 128.3, 120.0 (CH=*C*H_2_), 93.6 (C-1), 71.2 (C-5), 69.4 (C-4), 67.8 (C-3), 67.3 (C-2), 66.6 (O*C*H_2_CH=CH_2_); ES-HRMS calcd for C_32_H_26_O_10_NCl_3_Na 712.0520, found *m/z* 712.0545 [M+Na]^+^.

*((2S,3R)-2-Azido-3-benzyloxyicosanyl) 2,3,4-tri-O-benzoyl-β-d-galactopyranuronic acid, allyl ester* (**22a**)*.* Alcohol **16** (78 mg, 0.18 mmol) and imidate **11** (133 mg, 0.19 mmol) were dissolved in CH_2_Cl_2_ (6 mL) and stirred over 4Å MS for 20 min then cooled to 0 °C. To this TMSOTf (0.1M, 0.035 mmol, 0.35 mL) was added and the reaction stirred at 0 °C for 40 min. Solid NaHCO_3_ (30 mg) was added and the mixture stirred for 20 min and then filtered through celite, which was rinsed with CH_2_Cl_2_. The solvent was then removed under reduced pressure and flash chromatography (EtOAc-cyclohexane 1:4) gave **22a** (127 mg, 75%) as a yellow oil; R_f_ 0.50 (EtOAc-cyclohexane 2:5); [α]_D_ +76 (c 6.6 ,CHCl_3_); IR (film) cm^−1^: 3064, 2924, 2853, 2099, 1734, 1261, 1108; ^1^H-NMR (CDCl_3_, 500 MHz): δ 7.99 (2H, dd, *J =* 8.3 Hz, *J =* 1.1 Hz, aromatic H), 7.95 (2H, *J =* 8.3 Hz, *J =* 1.1 Hz, aromatic H), 7.81 (2H, *J =* 8.3 Hz, *J =* 1.1 Hz, aromatic H), 7.56 (1H, tt, *J =* 7.5 Hz, *J =* 1.2 Hz, aromatic H), 7.50 (1H, tt, *J =* 7.4 Hz, *J =* 1.2 Hz, aromatic H), 7.43 (1H, t, *J =* 7.5 Hz, aromatic H), 7.36 (8H, m, aromatic H), 7.26 (3H, m, aromatic H), 6.21 (1H, dd, *J =* 3.5 Hz, *J =* 1.2 Hz, H-4), 5.82 (1H, dd, *J =* 10.4 Hz, *J =* 7.9 Hz, H-2), 5.75 (1H, ddt, *J =* 17.2 Hz, *J =* 10.3 Hz, *J =* 6.0 Hz, C*H*=CH_2_), 5.63 (1H, dd, *J =* 10.4 Hz, *J =* 3.5 Hz, H-3), 5.23 (1H, dd, *J =* 17.2 Hz, *J =* 1.3 Hz, CH=C*H_2_*), 5.07 (1H, dd, *J =* 10.3 Hz, *J =* 1.0 Hz, CH=C*H_2_*), 4.91 (1H, d, *J =* 7.9 Hz, H-1), 4.58–4.64 (3H, overlapping signals, BnCH*H*, H-5, OC*H_2_*CH=CH_2_) 4.56 (1H, ddt, *J =* 13.0 Hz, *J =* 6.0 Hz, *J =* 1.0 Hz, OC*H_2_*CH=CH_2_), 4.46 (1H, d, 11.3 Hz, BnC*H*H), 4.25 (1H, dd, *J =* 10.7 Hz, *J =* 5.6 Hz, C*H*HO), 3.81 (1H, dd, *J =* 10.7 Hz, *J =* 5.0 Hz, CH*H*O), 3.70 (1H, dd, *J =* 10.7 Hz, *J =* 5.4 Hz, C*H*N_3_), 3.50 (1H, ddd, *J =* 3.5 Hz, *J =* 5.5 Hz, *J =* 8.0 Hz, C*H*OBn), 1.45 (2H, m, C*H_2_*), 1.27 (30H, s, 15 C*H_2_*), 0.88 (3H, t, *J =* 6.9 Hz, C*H_3_*); ^13^C-NMR (CDCl_3_, 125 MHz): δ 165.5, 165.4, 165.1, 165.0 (each C=O), 138.2, 133.4, 133.3, 133.2, 130.9, 130.0, 129.8, 129.7, 129.2, 128.9, 128.7, 128.5, 128.3 (3s), 128.0, 127.6, 119.7 (CH=*C*H_2_), 101.3 (C-1), 78.3 (*C*HOBn), 72.9 (C-5), 72.7 (Bn*C*H_2_), 71.3 (C-3), 69.2 (C-2), 69.0 (C-4), 68.9 (*C*H_2_O), 66.5 (O*C*H_2_CH=CH_2_), 63.1 (*C*HN_3_), 31.9, 30.7 (3s), 29.6 (2s), 29.5 (2s), 29.3, 26.9, 25.0, 22.6 (each *C*H_2_), 14.1 (*C*H_3_); ES-HRMS calcd for C_57_H_71_O_11_N_3_Na 996.4986, found *m/z* 996.4960 [M+Na]^+^.

*((2S,3R)-2-Azido-3-hydroxyicosanyl) 2,3,4-tri-O-benzoyl-α-D-galactopyranuronic acid, allyl ester* (**23a**)*.* The *β*-glycoside **22a** (125 mg, 0.129 mmol) was dissolved in CHCl_3_ (6 mL), to this TiCl_4_ (48 mg, 0.25 mmol, 27 µL) was added and the reaction was stirred at room temp for 3 h. The reaction mixture was poured onto satd NaHCO_3_-Et_2_O (1:9) and stirred for 30 min. The mixture was filtered through celite, which was rinsed with Et_2_O, the organic layer was decanted and dried over MgSO_4_. The solvent was removed under reduced pressure and then flash chromatography of the residue (EtOAc-cyclohexane 1:9) gave **23a** (114 mg, 99%) as a yellow oil; R_f_ 0.26 (EtOAc-cyclohexane 1:4); [α]_D_ +110 (c 5.7, CHCl_3_); IR (film) cm^−1^: 3521, 3068, 2924, 2853, 2098, 1732, 1265, 1094, 1026; ^1^H-NMR (CDCl_3_, 500 MHz): δ 8.00 (4H, t, *J =* 7.0 Hz, aromatic H), 7.81 (2H, dd, *J =* 8.4 Hz, *J =* 1.2 Hz, aromatic H), 7.59 (1H, t, *J =* 7.5 Hz, aromatic H), 7.51 (1H, t, *J =* 7.5 Hz, aromatic H), 7.45 (3H, m, aromatic H), 7.37 (2H, t, *J =* 7.8 Hz, aromatic H), 7.26 (2H, t, *J =* 7.8 Hz, Hz), 6.28 (1H, dd, *J =* 3.5 Hz, *J =* 1.4 Hz, H-4), 6.01 (1H, dd, *J =* 10.8 Hz, *J =* 3.5 Hz, H-3), 5.76 (1H, ddt, *J =* 17.2 Hz, *J =* 10.4 Hz, *J =* 6.1 Hz, C*H*=CH_2_), 5.70 (1H, dd, *J =* 10.8 Hz, *J =* 3.6 Hz, H-2), 5.61 (1H, d, *J =* 3.6 Hz, H-1), 5.23 (1H, dd, *J =* 17.2 Hz, *J =* 1.3 Hz, CH=C*H_2_*), 5.08 (1H, dd, *J =* 10.4 Hz, *J =* 0.9 Hz, CH=C*H_2_*), 4.96 (1H, d, *J =* 1.4 Hz, H-5), 4.62 (1H, dd, *J =* 12.7 Hz, *J =* 6.1 Hz, OC*H_2_*CH=CH_2_), 4.57 (1H, dd, *J =* 12.7 Hz, *J =* 6.1 Hz, OC*H_2_*CH=CH_2_), 4.12 (1H, dd, *J =* 10.6 Hz, *J =* 2.8 Hz, CH*H*O), 3.80 (1H, dd, *J =* 10.6 Hz, *J =* 6.8 Hz, C*H*HO), 3.69 (1H, m, C*H*OH), 3.48 (1H, td, *J =* 6.5 Hz, *J =* 2.8 Hz, C*H*N_3_), 1.80 (1H, d, *J =* 4.6 Hz, O*H*), 1.38 (4H, m, 2 C*H_2_*), 1.26 (s, 28H, 14 C*H_2_*), 0.88 (3H, t, *J =* 0.88 Hz, C*H_3_*); ^13^C-NMR (CDCl_3_, 125 MHz): δ 166.4, 165.9, 165.4, 165.1 (each C=O), 133.5 (2s), 133.2, 130.8, 129.9, 129.8, 129.7, 129.0 (2s), 128.9, 128.5, 128.4, 128.2, 119.9 (alkene CH_2_), 97.6 (C-1), 71.2 (*C*HOH), 69.8 (C-4), 69.3, 69.2, 68.5 (C-2), 67.8 (C-3), 66.5 (O*C*H_2_CH=CH_2_), 65.3 (*C*HN_3_), 33.7, 31.9, 29.7, 29.6, 29.5 (3s), 29.3, 26.9, 25.5, 22.6 (each *C*H_2_), 14.1 (*C*H_3_); ES-HRMS calcd for C_50_H_65_O_11_N_3_Na 906.4517, found *m/z* 906.4526 [M+Na]^+^.

*((2S,3R)-2-(N-Nonadecanoylamino)-3-hydroxyicosan-1-yl) 2,3,4-tri-O-benzoyl-α-D-galactopyran-uronic acid, allyl ester* (**24a**). Azide **23a** (110 mg, 0.125 mmol) was dissolved in CH_2_Cl_2_ (4 mL) and PBu_3_ (62 μL, 0.25 mmol) was added. The reaction was stirred at room temp for 1.5 h followed by the addition of H_2_O (2 mL) and MeOH (0.5 mL) and the reaction stirred for a further 2 h. The reaction was diluted with Et_2_O, washed with water, brine, dried over MgSO_4_, and the solvent removed under reduced pressure. The crude amine was take up in CH_2_Cl_2_ (4 mL) and cooled to 0 °C, to this DIEPA (48 mg, 0.37 mmol, 65 µL) and nonadecanoyl chloride (0.50 mL of 0.25 mM, 0.125 mmol) were respectively added, and the reaction allowed to attain room temp over 16 h. The reaction mixture was taken up in Et_2_O, washed with water, brine, dried over MgSO_4_ and the solvent was removed under reduced pressure. Flash chromatography (EtOAc-cyclohexane 1:4) gave **24a** (15 mg, 12%) as a white solid; R_f_ 0.18 (EtOAc-cyclohexane 2:5); IR (film) cm^−1^: 3309, 2917, 2850, 1731, 1650, 1266, 1094; ^1^H-NMR (CDCl_3_, 500 MHz): δ 8.01 (2H, d, *J =* 7.2 Hz, aromatic H), 7.96 (2H, d, *J =* 7.3 Hz, aromatic H), 7.82 (2H, d, *J =* 7.2 Hz, aromatic H), 7.61 (1H, t, *J =* 7.5 Hz, aromatic H), 7.54 (1H, t, *J =* 7.4 Hz, aromatic H), 7.46 (3H, m, aromatic H), 7.40 (2H, t, *J =* 7.8 Hz, aromatic H), 7.29 (2H, t, *J =* 7.9 Hz, aromatic H), 6.31 (1H, d, *J =* 8.1 Hz, NH), 6.26 (1H, dd, *J =* 3.4 Hz, *J =* 1.3 Hz, H-4), 5.96 (1H, dd, *J =* 10.7 Hz, *J =* 3.4 Hz, H-3), 5.76 (1H, ddt, *J =* 17.2 Hz, *J =* 10.3 Hz, *J =* 6.2 Hz, C*H*=CH_2_), 5.68 (1H, dd, *J =* 10.7 Hz, *J =* 3.7 Hz, H-2), 5.58 (1H, d, *J =* 3.7 Hz, H-1), 5.24 (1H, dd, *J =* 17.2 Hz, *J =* 1.2 Hz, CH=C*H_2_*), 5.10 (1H, dd, *J =* 10.3 Hz, *J =* 1.2 Hz, CH=C*H_2_*), 4.81 (1H, d, *J =* 1.3 Hz, H-5), 4.63 (1H, dd, *J =* 12.8 Hz, *J =* 6.2 Hz, OC*H_2_*CH=CH_2_), 4.57 (1H, dd, *J =* 12.8 Hz, *J =* 6.2 Hz, OC*H_2_*CH=CH_2_), 4.10 (1H, dd, *J =* 10.4 Hz, *J =* 2.2 Hz, CH*H*O), 4.00 (1H, m, C*H*OH), 3.83 (1H, dd, *J =* 10.4 Hz, *J =* 3.6 Hz, CH*H*O), 3.56 (1H, m, C*H*N), 2.39 (1H, brs, OH), 2.24 (2H, m, COC*H_2_*), 1.62 (4H, m, 2 C*H_2_*), 1.27 (60H, br s, 30 C*H_2_*), 0.89 (6H, t, *J =* 6.9 Hz, 2 C*H_3_*); ^13^C-NMR (CDCl_3_, 125 MHz): δ173.2, 166.1, 165.5 (2s), 165.1 (each C=O), 133.8, 133.6, 133.3, 130.8, 130.0, 129.8, 129.7, 128.9 (2s), 128.6 (2s), 128.5, 128.3, 120.0 (alkene *C*H_2_), 97.4 (C-1), 73.2 (*C*HOH), 69.7 (2s), 69.1 (C-5), 68.7 (C-2), 67.8 (C-3), 66.6 (O*C*H_2_CH=CH_2_), 52.1 (*C*HN), 36.8 (CO*C*H_2_), 34.9, 31.9, 29.8, 29.7 (4s), 29.6, 29.5 (2s), 29.4 (4s), 26.9, 25.9, 25.8, 22.7 (each *C*H_2_), 14.1 (2 × *C*H_3_). ES-HRMS calcd for C_69_H_104_O_12_N 1138.7559, found *m/z* 1138.7515 [M+H]^+^.

*((2S,3R)-2-(N-Nonadecanoylamino)-3-hydroxyicosan-1-yl)* *α-d-galactopyranuronic acid* (**5**). The protected lipid **24a** (8 mg, 7 µmol) was dissolved in *n*-PrOH (4 mL) and H_2_O_2_ (30%, 0.3 mL), and to this *n*PrONa-*n*PrOH (0.1 M, 60 µmol, 600 µmL) was added at a rate of 100 µL/h. After the addition was completed, the reaction mixture was then centrifuged at 15000 rpm for 15 min, the supernatant was removed and the precipitate washed twice with water. The precipitate was then lyophilised to give **5** (1.2 mg, 22%) as a white solid; ^1^H-NMR (CDCl_3_, 600 MHz): δ 4.93 (1H, d, *J =* 2.3 Hz, H-1), 4.26 (1H, s, H-5), 4.22 (1H, m), 3.88 (1H, m, CN*H*), 3.81 (1H, dd, *J =* 10.1 Hz, *J =* 3.0 Hz, H-2), 3.78 (3H, overlapping signals), 3.62 (1H, td, *J =* 7.2 Hz, *J =* 1.9 Hz, C*H*OH), 2.21 (2H, t, *J =* 7.6 Hz, C*H_2_*CO_2_), 1.64-1.53 (4H, m, C*H_2_*), 1.27 (60H, br s, C*H_2_*), 0.89 (6H, t, *J =* 7.0 Hz, C*H_3_*); ^13^C-NMR (CDCl_3_, 125 MHz): δ 175.1 (C=O), 99.9 (C-1), 72.0 (C-5), 71.0 (C-3), 71.0 (C*H*OH), 70.8, 68.9, 68.2, 53.7 (*C*HN), 36.5 (CO*C*H_2_), 36.7, 32.3, 30.0 (2s), 29.9, 27.8, 29.7 (2s), 26.3, 26.1, 23.0, 14.2.

*1-Nonadecyne* (**28**). Nonadec-1-ene **27** (1.96 g, 7.37 mmol) was dissolved in CHCl_3_ (15 mL) and bromine (1.44 g, 9.00 mmol) was added, and the reaction mixture was then stirred for 30 min at room temp. The solution was diluted with cyclohexane, washed with Na_2_S_2_O_4_, water, brine, dried over MgSO_4_, and concentrated under reduced pressure to give 1,2-dibromononadecane (3.1 g, 97%) as a white solid; R_f_ 0.51 (cyclohexane); IR (film) (cm^−1^): 2923, 2852, 1465, 1143; ^1^H-NMR (CDCl_3_, 400 MHz): δ 4.17 (1H, m, C*H*Br), 3.85 (1H, dd, *J =* 10.2 Hz, *J =* 4.4 Hz, CH*H*Br), 3.63 (1H, t, *J =* 10.0 Hz, C*H*HBr), 2.14 (1H, m, CH*H*CHBr), 1.78 (1H, m, C*H*HCHBr), 1.56 (2H, m), 1.27 (28H, s, CH_2_), 0.88 (3H, t, *J =* 6.8 Hz, C*H_3_*); ^13^C-NMR (CDCl_3_, 100 MHz): δ 53.2 (*C*HBr), 36.4 (*C*H_2_Br), 36.0, 31.9, 29.7 (2 s), 29.6, 29.5, 29.4 (2s), 28.8, 26.8, 22.7, 14.1 (*C*H_3_). 1,2-Dibromononadecane (2.00 g, 4.69 mmol) and KOH (2.67 g, 46.9 mmol) were dissolved in EtOH (40 mL) and heated at reflux for 48 h. The reaction mixture was then diluted with cyclohexane, washed with water, brine, dried over MgSO_4_, and the solvent was removed under reduced pressure. Flash chromatography (cyclohexane) gave **28** (0.91 g, 73%) as a white solid; R_f_ 0.51 (cyclohexane);IR (film) (cm^−1^): 3287, 2917, 2849, 1462, 908, 731, 630; ^1^H-NMR (CDCl_3_, 400 MHz): δ 2.16 (2H, td, *J =* 7.1 Hz, *J =* 2.6 Hz, HCCC*H_2_*), 1.89 (1H, t, *J =* 2.6 Hz, CH), 1.52 (2H, m), 1,43 (30H, s), 0.88 (3H, t, J = 6.7, C*H_3_*); ^13^C-NMR (CDCl_3_, 100 MHz): δ 84.5 (HC*C*), 68.0 (C*C*H), 32.0, 29.8, 29.7 (2s), 29.6, 29.5, 29.2, 28.8, 28.6, 27.0, 22.7, 18.4, 14.1 (*C*H_3_).

*((2S,3R)-2-(4-Heptadecyl-1H-1,2,3-triazolyl)-3-hydroxyicosan-1-yl) 2,3,4-tri-O-benzoyl-α-d-galacto-pyranuronic acid, allyl ester* (**30**) and *((2S,3R)-2-(5-heptadecyl-1H-1,2,3-triazolyl)-3-hydroxyicosan-1-yl) 2,3,4-tri-O-benzoyl-α-d-galactopyranuronic acid, allyl ester* (**31**). Azide **23b** (78 mg, 88.3 µmol) and alkyne 28 (46 mg, 177 µmol) were dried under vacuum. The reagents were taken up in toluene (1.5 mL), which was followed by the addition of copper(I) iodide (17 mg, 88 µmol), and the reaction mixture heated at reflux for 24 h. The reaction mixture was purified by flash chromatography to give (32 mg, 32%) as an inseparable 86:14 mixture of 30:31 (yellow solid); [α]_D_ +32.0 (c 1.0, CHCl_3_); IR (film) cm^−1^: 3406, 3070, 2919, 2850, 1733, 1452, 1264, 1108, 1069, 709; ^1^H-NMR for major isomer (CDCl_3_, 500 MHz): δ 7.93 (2H, dd, *J =* 8.4 Hz, *J =* 1.2 Hz, aromatic H), 7.91 (2H, dd, *J =* 8.4 Hz, *J =* 1.2 Hz, aromatic H), 7.88 (2H, dd, *J =* 8.4 Hz, *J =* 1.2 Hz, aromatic H), 7.52 (1H, m, aromatic H), 7.44 (2H, m, aromatic H), 7.37 (5H, m, aromatic H), 7.31 (2H, t, *J =* 7.8 Hz, aromatic H), 6.13 (1H, t, *J =* 9.8 Hz, H-3), 5.92 (1H, ddt, *J =* 17.0 Hz, *J =* 10.4 Hz, *J =* 6.1 Hz, C*H*=CH_2_) 5.67 (1H, t, *J =* 9.8 Hz, H-4), 5.41 (1H, d, *J =* 3.6 Hz, H-1), 5.30 (1H, dd, *J =* 9.8 Hz, *J =* 3.6 Hz, H-2), 5.18 (1H, dd, *J =* 17.1 Hz, *J =* 1.3 Hz, CH=C*H_2_*), 5.08 (1H, dd, *J =* 10.4 Hz, *J =* 0.9 Hz, CH=C*H_2_*), 4.56–4.64 (2H, overlapping signals), 4.48–4.54 (2H, overlapping signals), 4.30 (1H, dd, *J =* 11.1 Hz, *J =* 2.8 Hz, CH*H*O), 4.15 (1H, dd, *J =* 11.1 Hz, *J =* 6.8 Hz, C*H*HO), 4.05 (1H, br s, C*H*OH), 2.69 (1H, s, OH), 2.50 (1H, dt, *J =* 15.0 Hz, *J =* 7.9 Hz, C*H*H), 2.42 (1H, dt, *J =* 15.0 Hz, *J =* 7.8 Hz, C*H*H), 1.00–1.75 (64H, 32 x CH_2_), 0.88 (6H, t, *J =* 6.9 Hz, 2 x CH_3_); ^13^C-NMR (CDCl_3_, 125 MHz): δ 167.0, 165.6, 165.3, 165.1 (each C=O), 148.4 (N=*C*H), 133.6, 133.4, 133.3, 130.7, 129.9, 129.8, 129.7, 128.9, 128.8, 128.6 (2s), 128.4 (2s), 128.3, 121.2, 119.6 (alkene *C*H_2_), 97.0 (C-1), 71.4 (*C*HN), 71.2 (C-2), 69.8 (C-4), 69.5 (C-5), 69.3 (C-3), 67.8 (*C*H_2_O), 66.9 (O*C*H_2_CH=CH_2_), 64.9 (*C*HOH), 33.8, 31.9, 29.7 (2s), 29.6 (2s), 29.5 (2s), 29.4 (2s), 29.3, 25.6, 25.5, 22.7 (each *C*H_2_), 14.1 (*C*H_3_); ES-HRMS calcd for C_69_H_102_O_11_N_3_ 1148.7514, found *m/z* 1148.7570 [M+H]^+^.

*((2S,3R)-2-(4-Heptadecyl-1H-1,2,3-triazolyl)-3-hydroxyicosan-1-yl)* *α-d-galactopyranuronic acid* (**10**). The 86:14 mixture of protected glycolipids **30** and **31** (10 mg, 8.7 µmol) were dissolved in *n*-PrOH (2 mL) and H_2_O_2_ (30%, 0.2 mL), and to this *n*PrONa-*n*PrOH (500 µmL of 0.1 M, 50 µmol) was added at a rate of 80 µL/h. Water (5 mL) was then added to the reaction mixture and the solution centrifuged at 15000 rpm for 15 min, the supernatant was removed and the precipitate washed twice with water. The precipitate was then lyophilised to give a mixture of regioisomers (3.6 mg, 52%) where the 1,4-regioisomer **10** was the major product and there was also 12% of the 1,5-regioisomer. Analytical data for the major isomer **10**: ^1^H-NMR (CDCl_3_-CD_3_OD 2:1, 600 MHz): δ 7.64 (1H, s, triazole H), 4.85 (1H, d, *J =* 2.9 Hz, H-1), 4.59 (1H, br s, C*H*N), 4.10-4.20 (2H, m, C*H_2_*O), 4.03 (1H, td, *J =* 7.8 Hz, *J =* 3,4 Hz, C*H*OH), 3.88 (4H, br s, OH), 3.63–3.73 (2H, m, H-3, H-5), 3.40–3.45 (2H, m, H-2, H-4), 2.69 (2H, t, *J =* 7.8 Hz, C*H_2_*), 1.62–1.72 (2H, overlapping signals, C*H_2_*), 1.1–1.55 (60H, overlapping signals, C*H_2_*), 0.89 (6H, t, *J =* 6.8 Hz, 2 x C*H_3_*); ^13^C-NMR (CDCl_3_-CD_3_OD 2:1, 150 MHz): δ 176.5 (C=O), 130.1, 128.6 (triazole C and CH), 100.1 (C-1), 73.9, 72.6, 72.0, 70.7, 70.3, 68.2, 66.2, 33.8, 32.2, 30.0, 29.7, 29.6, 25.8, 25.7, 22.9, 14.2; ES-HRMS calcd for C_45_H_84_O_8_N_3_ 794.6258, found *m/z* 794.6276 [M-H]^−^.

*2,3,4-Tri-O-acetyl-α-thio-d-glucopyranosiduronic acid, methyl ester* (**15**). To a stirred solution of the β-thiol^10^ **34** (100 mg, 0.28 mmol) in CH_2_Cl_2_ (3 mL) was added TiCl_4_ (76 µL, 0.7 mmol) dropwise. The reaction mixture was stirred for 10 min at room temp and then cooled to 0 °C overnight, and CH_2_Cl_2_ and satd NH_4_Cl were then added, and the phases were separated and the aqueous phase was extracted with CH_2_Cl_2_. The combined organic layers were washed with water, brine, dried over MgSO_4_ and the solvent was removed to give an 8:1 mixture of **15** and **34** (65 mg, 65%) as a white foam. Analytical data for **15**: IR (film) cm^−1^: 2955, 2572, 1747, 1438, 1370, 1214, 1077, 1042; ^1^H-NMR (CDCl_3_, 500 MHz): δ 5.98 (1H, t, *J =* 5.7, H-1), 5.39 (1H, t, *J =* 9.0, H-3), 5.17 (1H, t, *J =* 8.9, H-4), 5.02 (1H, dd, *J =* 9.3, 5.2, H-2), 4.76 (1H, d, *J =* 9.2, H-5), 3.76 (3H, s, OCH_3_), 2.09 (3H, s), 2.06 (3H, s), 2.05 (3H, s) (each OAc), 2.02 (1H, s, SH); ^13^C-NMR (126 MHz, CDCl_3_) δ 169.5, 169.4, 167.7 (each C=O), 76.4 (C-1), 69.8 (C-2), 69.4 (C-5), 68.9 (C-4), 68.6 (C-3), 52.8 (OMe), 20.6, 20.5 (2s) (each OAc); ESI-HRMS calcd for C_13_H_17_O_9_S 349.0593, found *m/z* 349.0584 [M-H]^−^.

*Methyl ((4S,5R)-5-heptadecyl-2,2-dimethyl-3-t-butoxycarbonyl-oxazolidin-4-yl)methyl 2,3,4-tri-O-acetyl-thio-α-d-glucopyranosiduronate* (**44a**). Thiol 15 (155 mg, 0.44 mmol) was dissolved in dry DMF (3 mL) and cooled to 0 °C. Sodium hydride (16 mg of a 60% dispersion in mineral oil, 0.4 mmol) was added slowly and the reaction was stirred for 30 min. A solution of the bromide 18 (179 mg, 0.15 mmol) in DMF was then added dropwise and the mixture allowed to attain room temp and stirred overnight. EtOAc and water were added and the aqueous layer was washed with EtOAc. The combined organic layers were washed with water, brine, dried over MgSO_4_ and the solvent was removed. Flash chromatography (petroleum ether-EtOAc 4:1) gave **44a** (42 mg, 39%) as a colourless oil; [α]_D_ +60 (*c*, CHCl_3_); IR (film) cm^−1^: 2923, 2853, 1752, 1697, 1457, 1375, 1177, 1040; ^1^H-NMR (500 MHz, CDCl_3_) δ 5.60–5.80 (1H, overlapping signals, H-1), 5.41–5.31 (1H, overlapping signals, H-3), 5.24–5.14 (1H, overlapping signals, H-4), 5.03 (1H, dd, *J =* 9.8, 5.5, H-2), 4.72 (1H, overlapping signals, H-5), 3.90–4.10 (2H, overlapping signals, CHO, CHN), 3.74 (3H, s, OMe), 2.08–2.00 (9H, overlapping signals, OAc), 1.05–1.80 (40H), 0.88 (3H, t, *J =* 6.7, CH_3_); ^13^C-NMR (125MHz, CDCl_3_) δ 169.8, 169.5, 169.4, 167.9 (C=O), 92.5, 83.3 (C-1), 80.1, 76.6 (C-3'), 70.0 (C-2), 69.5 (C-3), 69.1 (C-4), 68.9 (C-5), 58.6 (C-2'), 52.9 (OMe), 32.1, 29.9, 29.7, 29.5, 28.6 (each CH_2_), 22.8, 20.8, 20.7 (2s), 14.3 (each CH_3_); ESI-HRMS calcd for C_41_H_71_NO_12_SNa 824.4595, found *m/z* 824.4586 [M+Na]^+^.

*Methyl ((2S,3R)-2-(N-nonadecanoylamino)-3-hydroxyicosan-1-yl) 2,3,4-tri-O-acetyl-thio-d-glucopyranosiduronate* (**46a**). *N*-Hydroxysuccinimide (0.76 g, 6. 6 mmol) and EDC (1.25 g, 6.6 mmol) were added to a solution of nonadecanoic acid (1.87 g, 6.6 mmol) in CH_2_Cl_2_ (50 mL) and the mixture was stirred overnight at room temp. The solvent was concentrated under reduced pressure and the resulting residue was dissolved in CH_2_Cl_2_. The solution was washed with water, dried over MgSO_4_ and concentrated under reduced pressure to yield **45** as a white solid (2.45 g, 98%). This intermediate was used without further purification; ^1^H-NMR (500 MHz, CDCl_3_) δ 2.83 (4H, s, O=CC*H_2_*C*H_2_*C=O), 2.60 (2H, t, *J =* 7.5, CH_2_C=O), 1.79–1.69 (2H, m, CH_2_), 1.39 (2H, dd, *J =* 14.9, 7.0, CH_2_), 1.27 (29H, s, each alkyl CH_2_), 0.88 (3H, t, *J =* 6.9, CH_3_); ^13^C-NMR (125 MHz, CDCl_3_) δ 169.1, 168.6 (each C=O), 31.9, 30.9, 29.6, 29.6, 29.6, 29.5, 29.3, 29.0, 28.8, 25.6, 24.6, 22.7 (each CH_2_), 14.1 (CH_3_). Compound **44a** (30 mg, 0.04 mmol) was taken up in formic acid (3 mL) and stirred vigorously for 30 min. Toluene (5 mL) was added and the solvents were removed under reduced pressure. The resulting residue was taken up in water and basified with solid NaHCO_3_. The mixture was then extracted into CH_2_Cl_2_, dried over MgSO_4_ and the solvent was removed under reduced pressure. The resulting residue was taken up in CH_2_Cl_2_ (2 mL) and DIPEA (22 µL, 0.13 mmol) was added. To this was added a solution of **45** (37 mg, 0.09 mmol) in CH_2_Cl_2_ (1.5 mL) and the mixture was stirred at room temp for 16 h. The reaction mixture was partitioned between EtOAc and satd NaHCO_3_. Phases were separated and the aqueous phase extracted into EtOAc. The combined organic phases were washed with brine, dried over MgSO_4_ and the solvent was removed under reduced pressure. Flash chromatography (petroleum ether-EtOAc 3:1) gave **46a** (19 mg, 57% over two steps) as a white solid; [α]_D_ +48 (c 1.0 in CHCl_3_); IR (film) cm^−1^: 3294 (br), 2917, 2850, 1751, 1650, 1467, 1219, 1043; ^1^H-NMR (500 MHz, CDCl_3_) δ 6.12 (1H, d, *J =* 8.5, NH), 5.69 (1H, d, *J =* 5.2, H-1), 5.29 (1H, t, *J =* 9.0, H-3), 5.21 (1H, t, *J =* 9.0, H-4), 4.99 (1H, dd, *J =* 9.0, 5.2, H-2), 4.74 (1H, d, *J =* 9.0, H-5), 4.01 (1H, br s, CHO), 3.79–3.73 (3H, m, OMe), 3.68 (1H, br s, CHN), 3.03 (1H, dd, *J =* 14.0, 8.0, SC*H*_2_), 2.84 (1H, dd, *J =* 14.0, 3.5, SC*H*_2_), 2.19 (2H, d, *J =* 4.3, CH_2_), 2.08, 2.04, 2.03 (each 3H, each s, each CH_3_), 1.62 (4H, s, 2 × CH_2_), 1.45 (2H, m, CH_2_), 1.32–1.22 (60H, overlapping signals, each CH_2_), 0.87 (6H, t, *J =* 6.9, 2 × CH_3_); ^13^C-NMR (125 MHz, CDCl_3_) δ 173.7, 169.8, 169.5, 169.4, 167.9 (each C=O), 83.5 (C-1), 73.6 (CHO), 70.0 (C-2), 69.2 (2s) (C-3 & C-5), 68.8 (C-4), 54.0 (CHN), 53.0 (OMe), 36.7, 34.0, 31.9 (each CH_2_), 29.7 (3s), 29.6, 29.4 (2s), 26.0, 25.7, 22.7 (each CH_2_), 20.7, 20.6 (2s), 14.1 (each CH_3_); ESI-HRMS calcd. for C_52_H_94_NO_11_S 940.6548, found *m/z* 940.6578 [M-H]^−^.

*Methyl ((2S,3R)-2-(N-nonadecanoylamino)-3-hydroxyicosan-1-yl) 2,3,4-tri-O-acetyl-thio-d-glucopyranosiduronate* (**8**). Protected lipid derivative **46a** (3.0 mg, 0.3 µmol) was dissolved in anhydrous EtOAc (200 µL) and LiI (15 mg, 0.11 mmol) was added. The reaction mixture was heated at 70 °C for 6 h. Upon cooling the reaction mixture was washed with H_2_O, brine, dried over MgSO_4_ and concentrated under reduced pressure. The resulting residue was then taken up in a guanidine-guanidinium nitrate solution (2 mL, 1M in CH_2_Cl_2_-MeOH 1:9) the reaction was stirred at room temp for 1 h. The reaction was neutralised by the addition of Amberlite® resin IR-20, filtered and the solvent was removed under reduced pressure. The crude product was purified using lipophilic Sephadex^®^ LH-20 to give the title compound (1.4 mg) as a white powder; ^1^H-NMR (500 MHz, CDCl_3_-MeOD 2:1) δ 5.21 (1H, d, *J =* 4.5), 4.31 (1H, br s), 3.78 (1H, br s), 3.58 (2H, br s), 3.45 (1H, s), 3.31–3.50 (2H, overlapping signals), 2.55–2.80 (2H, m, SCH_2_), 0.85-2.15 (66H), 0.65 (6H, br s, 2 CH_3_); ^13^C-NMR (125 MHz, CDCl_3_-MeOD 2:1) δ 175.5 (C=O), 87.6 (C-1), 73.5 (CHOH), 71.4 (C-2), 70.4 (C-3), 69.0 (C-5), 67.2 (C-4), 53.6 (CHN), 35.3, 31.3 (*C*H_2_S), 34.0, 29.4, 26.1, 26.0, 22.9, 22.7, 19.3 (each CH_2_), 14.1 (CH_3_); ESI-HRMS calcd for C_45_H_86_NO_8_S 800.6074, found *m/z* 800.6077 [M-H]^−^.

## 4. Conclusions

Glycolipids with *O*-, *S*- and *SO*_2_-linkages, analogous to antigen components of *Spingomonadacaece* have been prepared via the anomerisation of β-*O*-glycosides, β-*S*-glycosides or β-*S*-glycosyl thiols. This chemistry was more effective for the preparation of glucuronic acid or galacturonic acid derivatives than for glucose or galactose derivatives, consistent with a chelation induced anomerisation [37]. Although not always required, there can be an advantage to using benzoylated substrates in these reactions, as opposed to acetylated substrates [38]. The reasons for this are not fully understood. The synthesis of the uronic acid based glycosyl thiols from the β-precursor using TiCl_4_ is interesting as there are relatively few syntheses of α-glycosyl thiols reported to date [39] and such building blocks have wider potential, including *S*-disaccharide synthesis, for example. Triazole containing mimetics of the natural glycolipids were also prepared by CuAAC. The glycolipid antigens are being evaluated currently for their effects on iNKT cells.
